# Hospitalization for community-acquired febrile urinary tract infection: validation and impact assessment of a clinical prediction rule

**DOI:** 10.1186/s12879-017-2509-3

**Published:** 2017-06-06

**Authors:** Janneke E. Stalenhoef, Willize E. van der Starre, Albert M. Vollaard, Ewout W. Steyerberg, Nathalie M. Delfos, Eliane M.S. Leyten, Ted Koster, Hans C. Ablij, Jan W. van’t Wout, Jaap T. van Dissel, Cees van Nieuwkoop

**Affiliations:** 1 Department of Infectious Diseases, Leiden University Medical Center, C5-P, PO Box 9600, 2300 RC Leiden, the Netherlands; 2 Department of Public Health, Erasmus MC - University Medical Center Rotterdam, Rotterdam, The Netherlands; 3 Dept of Internal Medicine, Alrijne Hospital, Leiderdorp, The Netherlands; 4 Dept of Internal Medicine, MCH-Bronovo, The Hague, The Netherlands; 5 Dept of Internal Medicine, Groene Hart Hospital, Gouda, The Netherlands; 6 Dept of Internal Medicine, Alrijne Hospital, Leiden, The Netherlands; 7 Dept of Internal Medicine, Haga Hospital, The Hague, The Netherlands

**Keywords:** Community-acquired febrile urinary tract infection, Severity assessment, Prediction tool, Hospitalization

## Abstract

**Background:**

There is a lack of severity assessment tools to identify adults presenting with febrile urinary tract infection (FUTI) at risk for complicated outcome and guide admission policy. We aimed to validate the Prediction Rule for Admission policy in Complicated urinary Tract InfeCtion LEiden (PRACTICE), a modified form of the *pneumonia severity index*, and to subsequentially assess its use in clinical practice.

**Methods:**

A prospective observational multicenter study for model validation (2004–2009), followed by a multicenter controlled clinical trial with stepped wedge cluster-randomization for impact assessment (2010–2014), with a follow up of 3 months. Paricipants were 1157 consecutive patients with a presumptive diagnosis of acute febrile UTI (787 in validation cohort and 370 in the randomized trial), enrolled at emergency departments of 7 hospitals and 35 primary care centers in the Netherlands.

The clinical prediction rule contained 12 predictors of complicated course. In the randomized trial the PRACTICE included guidance on hospitalization for high risk (>100 points) and home discharge for low risk patients (<75 points), in the control period the standard policy regarding hospital admission was applied. Main outcomes were effectiveness of the clinical prediction rule, as measured by primary hospital admission rate, and its safety, as measured by the rate of low-risk patients who needed to be hospitalized for FUTI after initial home-based treatment, and 30-day mortality.

**Results:**

A total of 370 patients were included in the randomized trial, 237 in the control period and 133 in the intervention period. Use of PRACTICE significantly reduced the primary hospitalization rate (from 219/237, 92%, in the control group to 96/133, 72%, in the intervention group, *p* < 0.01). The secondary hospital admission rate after initial outpatient treatment was 6% in control patients and 27% in intervention patients (1/17 and 10/37; *p* < 0.001).

**Conclusions:**

Although the proposed PRACTICE prediction rule is associated with a lower number of hospital admissions of patients presenting to the ED with presumptive febrile urinary tract infection, futher improvement is necessary to reduce the occurrence of secondary hospital admissions.

**Trial registration:**

NTR4480 http://www.trialregister.nl/trialreg/admin/rctview.asp?TC=4480, registered retrospectively 25 mrt 2014 (during enrollment of subjects).

**Electronic supplementary material:**

The online version of this article (doi:10.1186/s12879-017-2509-3) contains supplementary material, which is available to authorized users.

## Background

The majority of adults presenting to hospital with an acute febrile illness suffer from respiratory or urinary tract infections [1, 2]. The course of infection may be unpredictable and fever may reflect the onset of sepsis with potential progression to septic shock and multi organ failure. However, adults with fever of bacterial origin usually present with a mild illness at emergency departments (ED) and respond favourably to antibiotic treatment. It thus appears that the vast majority of these patients can be managed safely as outpatients. In daily clinical practice the need for hospital-based treatment for febrile urinary tract infection (FUTI) is assessed on basis of history, comorbidity and on severity of local and vital signs.

For respiratory tract infection there are validated clinical rules to calculate the mortality risk, such as the *Pneumonia Severity Index* (PSI), which is used to provide guidance on decisions regarding treatment and hospital admission [3–5]. To date, there are no such rules to assess the risk of poor outcome in patients presenting with FUTI.

The risk of complicated course of FUTI increases with age and comorbidity, but the event rate of life-threatening complications is low [6–8]. Physicians tend to apply low thresholds for hospitalization, which suggests that many admissions may be avoidable [9, 10]. Therefore, clinical tools that predict prognosis in patients with FUTI are needed to identify those who benefit from hospital admission, and those who may safely be managed as outpatients.

The main predicting factors of mortality in the PSI are not specific for pneumonia such as age, co-morbidity and physical or laboratory signs of sepsis [3]. We therefore considered that this risk assessment might also apply for community-acquired infections other than pneumonia. As our focus was on the evaluation of a practical and bedside available prediction tool, we modified the PSI by erasing all the laboratory variables (Table 1) and changed the name in the *Prediction Rule for Admission policy in Complicated urinary Tract InfeCtion LEiden* (PRACTICE). We used data from a prospective observational multi-center cohort study that included 787 consecutive adults with febrile UTI between 2004 and 2009 to validate this PSI-derived prediction rule for complicated course in patients with FUTI (all details and methods are described in the Additional file 1). In this validation cohort, the PRACTICE score identified those at very low risk for 30-day mortality and ICU admission; the area under the curve (AUC) of the receiver operating characteristic curve for these outcomes indicated a good discriminatory power (AUC 30-day mortality: 0.91; AUC 30-day mortality or ICU admission: 0.84). The PRACTICE score was devided in 5 risk categories (see Additional file 1: Table S1), showing that patients with a PRACTICE score < 100 points (*n* = 636) had a very low risk (<2%) of adverse outcomes; yet 380 (60%) of those were hospitalized. Using a cut-off value of the PRACTICE score ≥ 100 resulted in a negative predictive value for 30-day mortality of 1.00 and for the composite endpoint ‘complicated course’ (30-day mortality, ICU admission or hospitalization >10 days) of 0.90. Because the cut-off point was chosen to identify low-risk patients, the positive predictive values (PPV) were low (PVV 0.12 and 0.39, respectively). We assumed that the PRACTICE is a good bedside clinical tool to distinguish patients with FUTI at low risk of complicated course who can be managed as outpatients.

**Table 1:** Prediction Rule for Admission policy in Complicated urinary Tract InfeCtion LEiden (PRACTICE)

Characteristic		Allocated points^a^
Demographic		
	Age (men)	Age (years)
	Age (women)	Age (years) - 10
Nursing home resident		+10
Comorbidity^b^		
	Malignancy	+30
	Congestive heart failure	+10
	Cerebrovascular disease	+10
	Liver cirrhosis	+20
	Renal disease	+10
Signs & Symptoms		
	Altered mental status	+20
	Respiratory rate ≥ 30/min	+20
	Systolic blood pressure < 90 mmHg	+20
	Pulse ≥125/min	+10
	Temperature ≥ 40 °C	+15

^a^A total score individual patient score is obtained by summing the points for each characteristic

^b^Malignancy is defined as any cancer except basal- or squamous-cell cancer of the skin that was active within the previous year of presentation. Congestive heart failure is defined as ventricular dysfunction for which the patient is prescribed medication and/or consults a hospital-based medical specialist. Cerebrovascular disease is defined as a history of stroke or transient ischemic attack. Liver disease is defined as a clinical diagnosis of cirrhosis. Renal disease is defined as a history of chronic renal disease

According to risk class the following recommendations will apply:

< 75 points strong recommendation towards home-based management

75–100 points consider home-based management

>100 points strong recommendation towards hospital admission

The aim of the present study is to validate the PRACTICE in a new prospective cohort to guide the need for hospitalization in patients with FUTI presenting at EDs, with the aim to reduce hospitalization rates without compromising clinical outcome.

## Methods

### Trial design

We performed a stepped wedge cluster-randomized trial involving consecutive patients presenting with a presumptive diagnosis of FUTI, at the EDs of 7 hospitals in the Netherlands, between January 2010 and June 2014 [11]. These centers also participated in the validation cohort study (see Additional file 1). All participating centers started with a control period, in which routine clinical practice with regard to hospitalization policy was applied. The intervention (use of the PRACTICE) was introduced at the participating centers sequentially, in random order. By the end of the allocation all sites, except one, used the PRACTICE to guide admission policy.

Inclusion criteria were age ≥ 18 years, fever (≥38.0 °C) and/or a history of fever or shaking chills within 24 h before presentation, at least one symptom of UTI (dysuria, perineal pain or flank pain) and a positive nitrite dipstick test or leucocyturia. Exclusion criteria were pregnancy, hemodialysis or peritoneal dialysis, a history of kidney transplantation or polycystic kidney disease. The study protocol was approved by the local Ethics Committee, and all participants signed an informed consent form prior to enrollment.

### Intervention and treatment

The PRACTICE score ranges from 8 to >125. Based on the validation cohort it was divided into three risk classes (low <75 points; intermediate 75–100 points; high >100 points) with corresponding recommendations regarding hospitalization policy (Table 1). During the control period, the decision to treat the patient at home or admission to hospital was made at the discretion of the ED physician. At the start of the intervention period the ED physicians were instructed to calculate the PRACTICE score and on that basis decide on hospital-based or home-based treatment. Preferably admission policy was done according to the guidance as described in Table 1, however, the attending physician was responsible for the final decision on treatment location.

Throughout the whole study period the antibiotic therapy was left at the discretion of the treating physician. According to local guidelines, outpatient treatment for FUTI consisted of a 10–14 day course of oral antimicrobials (first choice ciprofloxacin 500 mg b.i.d.) [12]. In case of risk factors for quinolone resistance a single dose of a long-acting parental antimicrobial, e.g. ceftriaxone or an aminoglycoside, at the initiation of therapy was advised while culture results were pending [13].

Admitted patients started with empirical antimicrobials intravenously according to local policy and were switched to an oral antibiotic based on antimicrobial sensitivity testing of the uropathogen cultured.

### Study procedures

Within 24–48 h of notification, qualified research nurses collected demographic and clinical data by reviewing the medical record completed with an interview by telephone or in person, using a standardized questionnaire. A midstream-catch urine culture and a set of blood cultures were taken before commencement of antimicrobial therapy. All patients were contacted in person 3–4 days and 28–32 days after enrollment, and contacted by phone at day 13–15 and day 84–92, to assess clinical outcome. Urine culture was repeated at the 28–32 day follow-up visit. In case of (re) admission during the study period, related data were obtained from the medical record and interview. In case a patient was lost to follow up, survival and readmission were assessed by inquiry with the patient’s primary care physicians, hospital chart and/or local governmental mortality registries.

Urine and blood cultures were performed using standard microbiological methods at local certified laboratories. Data collection of patients included during the validation period was identical (see Additional file 1).

### Endpoints

The primary endpoints were primary hospital admission rate (the percentage of patients who were directly admitted to hospital) and secondary hospital admission rate (the percentage of patients who needed to be hospitalized for FUTI after initial home-based treatment). Secondary outcome measures were 30- and 90-day all-cause mortality rate, ICU admission rate, the total number of hospitalization days over a 3-month follow-up and clinical- and microbiological cure rate through the 10- to 18-day post-treatment visit. Clinical cure was defined as being alive with absence of fever and resolution of UTI symptoms (either absence of symptoms or at least 2 points improvement on a 0 through 5 points severity score), without additional antimicrobial therapy for relapse of UTI [14]. Bacteriologic cure was defined as eradication of the study entry uropathogen with no recurrence of bacteriuria (pathogen growth <10^4^ cfu/mL in women or <10^3^ cfu/mL in men combined with disappearance of leucocyturia) [15].

A Data Safety Monitoring Board (DSMB) monitored the study and prescheduled interim analyses were performed according to predefined stopping rules. For the analysis of secondary hospital admission only low risk patients PRACTICE-score = < 100 points were considered.

### Definitions

UTI in men, postmenopausal women and in women with any structural or functional abnormality of the urinary tract were considered ‘complicated’ whereas in all others it was considered ‘uncomplicated’ UTI [13, 15]. Comorbidity was defined as the presence of any urinary tract disorder, heart failure, cerebrovascular disease, renal insufficiency, diabetes mellitus, malignancy or chronic obstructive pulmonary disease.

### Statistical analysis

The primary endpoints were analyzed on the intention-to-treat (ITT) and per-protocol (PP) population. Evaluable patients for ITT analysis included all patients who met the inclusion criteria and had at least 1 follow up visit. The PP population consisted of cases in which PRACTICE-hospitalization recommendations were actually followed in the intervention period and all cases in the control period. Binomial or categorical outcome measures were analyzed using Chi-square tests (Pearson’s or Fisher’s). Risk difference with 95% confidence interval (CI) was used to compare the differences of categorical outcomes. Tests of significance were at 0.05 level, two-tailed, for primary hospital admission rate.

A study sample size of 326 patients in both arms was calculated on the basis of secondary hospital admission rate, which was estimated to be approximately 5%, based on our previous study on FUTI [16], to have a power of 90% to show that the secondary admission rate in the intervention period (PRACTICE-guided management) is at least as low as the control period. As we were only interested for non-inferiority and not in equivalence in secondary hospital admission rate, the sample size calculation was based on a one-tailed alpha of 0.025. This implies that the 90% CI of a two-tailed Chi-square test should not cross the predefined risk difference of 2.5% higher secondary admission rates. All analyses were performed using SPSS 20.0 (SPPS Inc., USA).

## Results

### Study participants

**Fig. 1:**
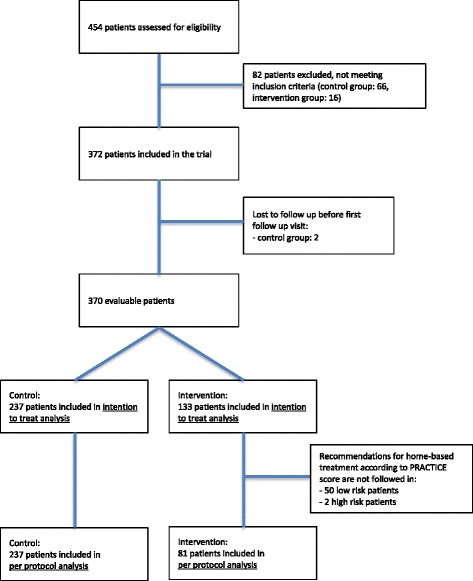
Patient inclusion flow chart

A total of 370 patients was included, 237 in the control period and 133 in the intervention period (see the flowchart in Fig. 1). In the ITT-population, baseline demographic characteristics were similar in the two groups (Table 2), except for a difference in history of cerebrovascular and chronic renal disease. Patients in who PRACTICE recommendations were followed (the PP-analysis) were significantly older, had more comorbidity and more often suffered complicated UTI than control patients (Table 2).

**Table 2:** Patients’ demographics

	Control group	Intervention group		*p*	
	ITT = PP	ITT	PP	Control vs ITT	Control vs PP
	*n* = 237	*n* = 133	*n* = 81		
Age in years; median, (IQR)	60 (30)	61 (34)	71 (26)	ns	<0,01
Sex – female	148 (62)	74 (56)	33 (41)	ns	<0,01
Febrile uncomplicated UTI	54 (23)	30 (23)	9 (11)	ns	0,02
Antimicrobial treatment at inclusion	90 (38)^a^	44 (33)	22 (27)	ns	ns
Urologic history					
Present urinary catheter	17 (7)	9 (7)	8 (10)	ns	ns
History of urinary tract disorder^a^	73 (31)	33 (25)	29 (36)	ns	ns
Recurrent UTI^b^	30 (13)^c^	11 (9)^c^	5 (7)	ns	ns
Comorbidity					
Any	124 (52)	77 (58)	57 (70)	ns	<0,01
Diabetes mellitus	36 (15)	29 (22)	25 (31)	ns	<0,01
Malignancy	13 (5)	11 (8)	10 (12)	ns	ns
Heart failure	32 (13)	12 (9)	11 (14)	ns	ns
Cerebrovascular disease	17 (7)	20 (15)	18 (22)	0.02	<0,01
Cirrhosis	1 (0)	2 (1)	1 (1)	ns	ns
Renal insufficiency	12 (5)	20 (15)	18 (22)	<0.01	<0,01
Immunocompromised	19 (8)	10 (8)	5 (6)	ns	ns

Data are presented as n (%) unless otherwise stated. ITT intention to treat analysis, PP per protocol analysis, IQR interquartile range, ns not significant (at 0,05 level), UTI urinary tract infection. ^a^Urinary tract disorder: presence of any functional or anatomical abnormality of the urinary tract excluding the presence of a urinary catheter. ^b^Recurrent UTI: two or more episodes in the last 6 months or three or more episodes of UTI in the last year. ^c^UTI history was unknown in 13 subjects in control period and 6 subjects in intervention period

Fifteen patients who were included in the study by ED-physicians did not completely meet the predefined inclusion criteria, but discharge diagnosis as concluded by the attending physician was FUTI in all cases. On hospital presentation, ten of these patients had no specific symptoms of UTI, 8 of these 10 patients had cultures of blood (3) and/or urine (5) positive with significant growth of an uropathogen, 2 had negative urine cultures, and 1 of them used antibiotics at inclusion. The other 5 patients did not have or report fever at inclusion, 1 of them was on TNFα-inhibitors.

Follow up was not completed in 37 patients in the control group and in 13 patients in the intervention group. Based on review of medical charts and governmental records these patients were all alive and without secondary admission, and included as such in the analysis.

### Cultures

The results of urine cultures, performed in 347 (93%) patients are shown in Table 3; 125 (36%) urine cultures were either sterile or contaminated of which 65% were obtained during antibiotic (pre)treatment. Blood cultures, performed in 357 (96%) patients, revealed bacteremia in 97 (27%) cases (Table 3). Rate of bacteremia was similar in intervention and control group.

**Table 3:** Bacteria isolated from baseline cultures

		Control period	Intervention period
		*n* = 237	*n* = 133
Urine cultures			
	*Escherichia coli*	126 (56)	51 (42)
	*Klebsiella spp*	12 (5)	7 (6)
	*Proteus spp*	5 (2)	3 (2)
	*Enterococcus spp*	3 (1)	-
	*Pseudomonas aeruginosa*	-	1 (1)
	*Staphylococcus aureus*	1 (0)	1 (1)
	Other	7 (3)	6 (5)
	Contaminated / mixed flora	26 (12)	24 (20)
Total positive urine cultures		154/225 (68)^a^	69/122 (57)^a^
Blood cultures			
	*Escherichia coli*	56 (25)	21 (68)
	*Klebsiella spp*	4 (6)	4 (13)
	*Proteus spp*	-	1 (3)
	*Enterobacter spp*	1 (1)	-
	*Pseudomonas aeruginosa*	1 (1)	-
	*Staphylococcus aureus*	1 (1)	2 (6)
	*Beta haemolytic streptococcus*	1 (1)	2 (6)
	*Citrobacter spp*	1 (1)	-
	*Bacteroides fragilis*	1 (1)	-
	*Salmonella paratyphi*	-	1 (3)
Total positive blood cultures		66/228 (29)^b^	31/129 (24)^b^

Data are presented as n (%). ^a^Urine cultures were not performed in 12 patients in the control period and 11 patients in the intervention period. ^b^Blood cultures were not obtained in 9 patients in the control period and 4 patients in the intervention period

### Outcome

The mean PRACTICE scores in the control and intervention groups (ITT analysis) were 62 (95% CI: 57.7 to 65.4) and 64 (95% CI: 58.3 to 69.7), respectively. Mean PRACTICE score in the PP population was 76 (95% CI: 69.0 to 83.3; *p* < 0,01).

Use of the PRACTICE significantly reduced primary hospitalization rate, 96 (72%) patients in the intervention group were admitted in the hospital versus 219 (92%) in the control period (*p* < 0.01) (Table 4). The hospitalization rate was further reduced to 57% in the PP population.

**Table 4:** Patients’ outcomes

		Control period	Intervention period ITT	Intervention period PP
		*n* = 237	*n* = 133	*n* = 81
Hospitalization				
	Primary hospitalization	219 (92)*	96 (72)*	46 (57)*
	Low risk	136	50	0
	Intermediate risk	58	29	29
	High risk	25	17	17
	Secondary admission (all risk classes)	2/18 (11)	10/37 (27)	10/35 (29)
	Low risk	1/17	6/29	6/29
	Intermediate risk	0/0	4/6	4/6
	High risk	1/1	0/2	0/0
	Need for ICU admission	8 (3)	1 (1)	1 (1)
	Hospital admission >10 days	15 (6)	10 (8)	9 (11)
	Total number of hospitalization days in 90 days of follow up [median, CI]	5 [5,6–7,0]	4 [4,4–6,7]	4 [4,2–7,6]
Bacteremia		66/228 (29)	31/129 (24)	21/77 (27)
Mortality				
	30-day all-cause mortality	3 (1)	3 (2)	2 (2)
	90-day all-cause mortality	7 (3)	5 (4)	4 (5)
Cure at day 30				
	Clinical cure	182/209 (87)	98/121 (80)	59/73 (81)
	Microbiological cure	170/190 (89)	107/113 (95)	61/65 (94)

Data are presented as n (%) unless otherwise stated. CI confidence interval, ITT intention to treat analysis, PP per protocol analysis, ICU intensive care unit. *p = < 0.001

The attending physician overruled the PRACTICE rule in 50 out of 153 patients categorized as low risk, who were admitted to the hospital because of ‘sick appearance’ (*n* = 9), severe flank pain (*n* = 2), antibiotic treatment at presentation (*n* = 7), comorbidity (*n* = 5), nausea (*n* = 3), uncertain diagnosis (*n* = 4), unknown (*n* = 7) or other reasons (*n* = 13). On the other hand, two patients categorized as high risk were treated at home because they insisted on home based treatment.

The median number of hospitalization days over a 3-month follow-up was 5 days (95% CI 5.6 to 7.0) vs 4 days (95% CI 4.4 to 6.7) for the control and intervention period, respectively.

Clinical and microbiological cure on day 30 did not differ significantly between both groups (Table 4).

The clinical outcomes according to risk class are outlined in Table 5.

**Table 5:** Clinical outcome of febrile urinary tract infection according to PRACTICE risk class; control and intervention groups combined

PRACTICE score (points)	Low risk			Intermediate risk			High risk			Total
	Class I-II			Class III			Class IV-V			
	(<75)			(76–100)			(>100)			
	Control	Intervention	All	Control	Intervention	All	Control	Intervention	All	
No. of patients	153	79	232	58	35	93	26	19	45	370
Clinical outcome										
30-day mortality, %	0	0	0	3 (5)	1 (3)	4 (4)	3 (11)	2 (10)	5 (11)	9 (2)
90-day mortality, %	0	0	0	3 (5)	3 (9)	6 (6)	4 (15)	2 (10)	6 (13)	12 (3)
ICU admission, %	3 (2)	0	3 (1)	2 (3)	0	2 (2)	3 (11)	1 (5)	4 (9)	9 (2)
Length of hospital stay										
Median no. of days [IQR]	4.0 [2]	3.0 [4]	4.0 [3]	6.0 [4]	4.0 [4]	5.0 [4]	6.5 [4]	6.0 [6]	6.0 [4]	5.0 [3]

Data are presented as n (%) unless otherwise stated, *IQR* interquartile range, *ICU* intensive care unit

### Safety

In the control period, 18 patients were treated at home (1 high risk and 17 low risk patients), of which 1 low risk patient was admitted 5 days after start of home treatment because of flank pain shown to be due to renal vein thrombosis.

Of the 37 patients in the intervention group who received initial home-based treatment (29 low risk, 6 intermediate risk and 2 high risk patients), 10 patients (27%) had a secondary hospital admission. These 10 patients (7 females; median age 61, range 18–85 years) had a low or intermediate risk for adverse events according to the PRACTICE-score (6 low, 4 intermediate), and were treated with oral ciprofloxacin (*n* = 9) or amoxicillin-clavulanic-acid (*n* = 1). Four out of 10 patients consulted the ED for re-evaluation on their own initiative because of worsening of symptoms such as fever or nausea. Six patients (60%) were contacted by phone by the treating physician to return to the hospital because of positive results of blood cultures, which grew *Escherichia coli* (*n* = 2, both ciprofloxacin sensitive), *Salmonella paratyphi* (*n* = 1), *Staphylococcus aureus* (*n* = 1) and Streptococcus Lancefield group A (*n* = 1) and G (*n* = 1). Median hospital stay was 2 days (range 1–14 days). In none of these secondary admissions intensive care treatment was required, and no complications were noted.

The first interim analysis, that took place after inclusion of 133 patients in the intervention group, showed an absolute risk difference in secondary hospital admission rate between intervention and control cohort of 23% (10/35 (29%) subjects in the intervention cohort, vs 1/17 (6%) in the control group). Because the difference in secondary admission rate exceeded the predefined stopping criterion of 20%, the DSMB advised to stop the trial.

## Discussion

We assessed the clinical use of a prediction rule, the PRACTICE, that stratifies patients presenting with FUTI into three risk groups for short-term mortality or admission to the ICU, and is based on bed-side available patient characteristics. Our hypothesis that the use of this prediction rule would reduce hospitalization rate was confirmed in this study, as shown by a 20% absolute reduction. The impact of the PRACTICE on admission policy could have been bigger, because in 33% of low risk patients PRACTICE-recommendations were overruled by the attending physician, possibly because of unfamiliarity with the decision rule. Patients in the PP population were older, had more comorbidity and thus a higher PRACTICE score, reflecting the fact that physicians were more likely to follow PRACTICE guidance when admission was recommended. The secondary admission rate of 29% exceeded the predefined stopping criterion (of a 20% absolute increase over that in the control group), and the study was stopped accordingly.

This real world study underlines the importance of the validation of clinical prediction rules in a new cohort to ensure its predictive value and usefulness in clinical setting, but there are some limitations.

The PRACTICE was adapted from the *Pneumonia Severity Index* (PSI). Selecting candidate predictors for prognostic modelling is generally done by logistic regression analysis. In order to have sufficient power, as a rule of thumb, we need at least ten outcomes per candidate predictor [17]. Predicting 30-day mortality rate of FUTI, which was estimated to be 2–5%, and considering analysis of 20 candidate predictors this implies a sample size of at least 4000–10,000 patients to obtain sufficient power. Based on previous studies, we realized such a large prospective study would be infeasible. Since the PRACTICE score was validated in a prospectively collected broad population of 787 patients and its impact was subsequently analyzed in a randomized intervention trial, our study was conducted according to guidelines for development of clinical prediction rules [18, 19]. As the PRACTICE predicts the composite outcome of complicated course (30-day mortality, ICU-admission and prolonged hospitalisation), according to the rule of thumb (one predictor for 10 or more outcomes), the validation cohort has sufficient power for reliable statistical analyses [17].

The trial was stopped because of safety concerns, since secondary hospital admission reached our predefined stopping rule. We note that all secondary admitted patients were discharged after a short and uncomplicated hospital stay. Two readmissions because of *E coli* bacteremia might have been avoided, because ciprofloxacin has been shown to be equally effective orally as intravenously in bacteremic UTI [20]. Among secondary admissions were patients with primary bacteremia caused by salmonella, staphylococci and streptococci, in whom presenting aspecific symptoms, e.g. fever and back pain, were mistaken for pyelonephritis, and sent home. Apparently, these patients were ‘misdiagnosed’ at first consultation as having FUTI, and subsequently were treated for other diagnoses at secondary admission. We included these patients in our analysis because the attending physicians at the EDs enrolled the patients in the current trial on a presumptive diagnosis of FUTI and we believe that these diagnostic errors reflect every day patient care [21].

Acute pyelonephritis and urosepsis are common conditions seen in the ED, and it is of importance to be aware that other unusual diseases can mimic its general symptoms. Other studies support our observation that the accuracy of UTI diagnosis may be suboptimal in the ED [22, 23].

Apparently the diagnosis of FUTI is not as straightforward as the diagnosis of pneumonia, where the presence of an infiltrate on chest X-ray is both definitive and confirmative and clinical decision rules such as the PSI have been implemented successfully in daily practice [3]. The PSI was derived from a large cohort of >14,000 patients and validated in almost 40,000 patients, and studies prospectively addressing its use in clinical practice found secondary admission rates of 4–9% [24–27]. The fact that we found higher secondary admission rates in FUTI, might also be explained by a different pathway leading to failure of home treatment in these two infections. Whereas respiratory distress is probably the main cause of secondary hospitalization of pneumonia patients; unability to take oral medication and need for volume resuscitation is more important for FUTI patients. These factors might be underrepresented in the composite outcome of complicated course of FUTI as predicted by the PRACTICE.

Differences in validation and intervention trial cohorts in this study might have attributed to the difference in secondary admission rate. In the historical cohort patients were recruited not only in EDs, but (a minority) also in the practice of general practitioners. The main difference with the historical cohort is the higher percentage of complicated UTI (or in some cases, an alternative diagnosis made on basis of blood culture findings) in the current cohort, which cannot be explained by a difference in sex or age. Other demographic parameters and outcome such as ICU admissions and mortality were comparable in the historical and current cohort.

Our patient group reflects the daily practice of patients presenting with community acquired FUTI, as both men and women, and patients with comorbidity were included. A previous study on women with acute pyelonephritis identified factors associated with hospital admission using a risk stratification model [28]. Age > 65 years, chills, segmented neutrophils >90%, creatinine >1.5 mg/dL, CRP >10 mg/dL and albumin 3.3 g/dL were independent risk factors for patient admission. Since details on mortality or complications are not given, no conclusion can be made on the actual risk for poor outcome. Furthermore, this model was not validated in a prospective cohort. In contrast, our PSI derived predictor variables can be readily assessed at the bedside level on the basis of history and physical examination.

How can the prediction rule for admission policy be optimized? The cut-off value of 75 points had a negative predictive value for predicting 30-day mortality of 100% in the intervention cohort. Lowering the threshold for admission policy in the intervention phase would hypotheticly have led to a hospitalization rate of 77% (102/133), but would still have resulted in a secondary hospitalization rate of 19% (6/31). The effect of the acute host response might be underrepresented in the PRACTICE, because it is based on the 30-day mortality in the validation cohort. Prognosis of the patient presenting with severe febrile illness consist of two factors. Firstly, the severity of the acute host response to the infection and inflammatory cascade eventually leading to shock and multi organ failure is best reflected by the hyperacute mortality. Secondly, the patients general health condition, mainly defined by age and comorbidity, that determines the 30-day mortality in patients who survive the first days of illness. Addition of a plasma biomarker reflecting the severity of sepsis, such as procalcitonin or midregional pro-adrenomedullin [29], might improve the decision rule in identifying patients who benefit from hospital-based treatment in the acute phase and lower the secondary admission rate. Furthermore, improved diagnosis of UTI is necessary to ensure safe implementation of prediction tools regarding clinical decision making.

## Conclusion

Implementation of the PRACTICE rule could decrease the number of hospital admissions of patients presenting to the ED with febrile urinary tract infection by 20%, at the expense of a high secondary admission rate.

## Additional file

Additional file 1: Supplementary data_PRACTICE validation cohort. (DOCX 38 kb)

## Abbreviations

AUC: Area under the curve
CI: Confidence interval
DSMB: Data safety monitoring board
ED: Emergency department
FUTI: Febrile urinary tract infection
ICU: Intensive care unit
ITT: Intention-to-treat
PP: Per-protocol (PP)
PRACTICE: Prediction Rule for Admission policy in Complicated urinary Tract InfeCtion Leiden
PSI: Pneumonia Severity Index
UTI: Urinary tract infection

## References

[1] van Dissel JT, Van LP, Westendorp RG, Kwappenberg K, Frolich M (1998). Anti-inflammatory cytokine profile and mortality in febrile patients. Lancet.

[2] Marco CA, Schoenfeld CN, Hansen KN, Hexter DA, Stearns DA, Kelen GD (1995). Fever in geriatric emergency patients: clinical features associated with serious illness. Ann Emerg Med.

[3] Fine MJ, Auble TE, Yealy DM, Hanusa BH, Weissfeld LA, Singer DE (1997). A prediction rule to identify low-risk patients with community-acquired pneumonia. N Engl J Med.

[4] Lim WS, van der Eerden MM, Laing R, Boersma WG, Karalus N, Town GI (2003). Defining community acquired pneumonia severity on presentation to hospital: an international derivation and validation study. Thorax.

[5] Charles PG, Wolfe R, Whitby M, Fine MJ, Fuller AJ, Stirling R (2008). SMART-COP: a tool for predicting the need for intensive respiratory or vasopressor support in community-acquired pneumonia. Clin Infect Dis.

[6] Foxman B, Klemstine KL, Brown PD (2003). Acute pyelonephritis in US hospitals in 1997: hospitalization and in-hospital mortality. Ann Epidemiol.

[7] Efstathiou SP, Pefanis AV, Tsioulos DI, Zacharos ID, Tsiakou AG, Mitromaras AG (2003). Acute pyelonephritis in adults: prediction of mortality and failure of treatment. Arch Intern Med.

[8] Buonaiuto VA, Marquez I, De TI, Joya C, Ruiz-Mesa JD, Seara R (2014). Clinical and epidemiological features and prognosis of complicated pyelonephritis: a prospective observational single hospital-based study. BMC Infect Dis.

[9] Ramakrishnan K, Scheid DC (2005). Diagnosis and management of acute pyelonephritis in adults. Am Fam Physician.

[10] Rhee JE, Kim K, Lee CC, Kang J, Lee JW, Shin JH (2011). The lack of association between age and diabetes and hospitalization in women with acute pyelonephritis. J Emerg Med.

[11] Brown C, Hofer T, Johal A, Thomson R, Nicholl J, Franklin BD (2008). An epistemology of patient safety research: a framework for study design and interpretation. Part 2. Study design. Qual Saf Health Care.

[12] van Asselt KM, Prins JM, van der Weele GM, Knottnerus BJ, Van PB GSE (2013). Unambiguous practice guidelines on urinary tract infections in primary and secondary care. Ned Tijdschr Geneeskd.

[13] Gupta K, Hooton TM, Naber KG, Wullt B, Colgan R, Miller LG (2011). International clinical practice guidelines for the treatment of acute uncomplicated cystitis and pyelonephritis in women: a 2010 update by the Infectious Diseases Society of America and the European Society for Microbiology and Infectious Diseases. Clin Infect Dis.

[14] van Nieuwkoop C, van’t Wout JW, Assendelft WJ, Elzevier HW, Leyten EM, Koster T (2009). Treatment duration of febrile urinary tract infection (FUTIRST trial): a randomized placebo-controlled multicenter trial comparing short (7 days) antibiotic treatment with conventional treatment (14 days). BMC Infect Dis.

[15] Rubin RH, Shapiro ED, Andriole VT, Davis RJ, Stamm WE (1992). Evaluation of new anti-infective drugs for the treatment of urinary tract infection. Infectious Diseases Society of America and the Food and Drug Administration. Clin Infect Dis.

[16] van Nieuwkoop C, van’t Wout JW, Spelt IC, Becker M, Kuijper EJ, Blom JW (2010). Prospective cohort study of acute pyelonephritis in adults: safety of triage towards home based oral antimicrobial treatment. J Inf Secur.

[17] Steyerberg EW, Eijkemans MJ, Harrell FE, Habbema JD (2001). Prognostic modeling with logistic regression analysis: in search of a sensible strategy in small data sets. Med Decis Mak.

[18] McGinn TG, Guyatt GH, Wyer PC, Naylor CD, Stiell IG, Richardson WS (2000). Users’ guides to the medical literature: XXII: how to use articles about clinical decision rules. Evidence-based medicine working group. JAMA.

[19] Steyerberg EW, Moons KG, van der Windt DA, Hayden JA, Perel P, Schroter S (2013). Prognosis research strategy (PROGRESS) 3: prognostic model research. PLoS Med.

[20] Mombelli G, Pezzoli R, Pinoja-Lutz G, Monotti R, Marone C, Franciolli M (1999). Oral vs intravenous ciprofloxacin in the initial empirical management of severe pyelonephritis or complicated urinary tract infections: a prospective randomized clinical trial. Arch Intern Med.

[21] Graber ML (2013). The incidence of diagnostic error in medicine. BMJ Qual Saf.

[22] Caterino JM, Ting SA, Sisbarro SG, Espinola JA, Camargo CA (2012). Age, nursing home residence, and presentation of urinary tract infection in U.S. emergency departments, 2001-2008. Acad Emerg Med.

[23] Gordon LB, Waxman MJ, Ragsdale L, Mermel LA (2013). Overtreatment of presumed urinary tract infection in older women presenting to the emergency department. J Am Geriatr Soc.

[24] Marrie TJ, Lau CY, Wheeler SL, Wong CJ, Vandervoort MK, Feagan BG (2000). A controlled trial of a critical pathway for treatment of community-acquired pneumonia. CAPITAL study investigators. Community-acquired pneumonia intervention trial assessing Levofloxacin. JAMA.

[25] Atlas SJ, Benzer TI, Borowsky LH, Chang Y, Burnham DC, Metlay JP (1998). Safely increasing the proportion of patients with community-acquired pneumonia treated as outpatients: an interventional trial. Arch Intern Med.

[26] Renaud B, Coma E, Labarere J, Hayon J, Roy PM, Boureaux H (2007). Routine use of the pneumonia severity index for guiding the site-of-treatment decision of patients with pneumonia in the emergency department: a multicenter, prospective, observational, controlled cohort study. Clin Infect Dis.

[27] Jo S, Kim K, Jung K, Rhee JE, Cho IS, Lee CC (2012). The effects of incorporating a pneumonia severity index into the admission protocol for community-acquired pneumonia. J Emerg Med.

[28] Kang C, Kim K, Lee SH, Park C, Kim J, Lee JH (2013). A risk stratification model of acute pyelonephritis to indicate hospital admission from the ED. Am J Emerg Med.

[29] van der Starre WE, Zunder SM, Vollaard AM, Van NC SJE, Delfos NM (2014). Prognostic value of pro-adrenomedullin, procalcitonin and C-reactive protein in predicting outcome of febrile urinary tract infection. Clin Microbiol Infect.

