# Supplementary material for: Hospitalization for community-acquired febrile urinary tract infection: validation and impact assessment of a clinical prediction rule
Source: BMC Infect Dis. 2017 Jun 6;17:400. doi: 10.1186/s12879-017-2509-3 (PMC5461732; doi:10.1186/s12879-017-2509-3)
Supplement: Additional file 1: — Supplementary data_PRACTICE validation cohort. (DOCX 38 kb) [file 12879_2017_2509_MOESM1_ESM.docx]

**SUPPLEMENTARY DATA**

**Practice validation cohort**

**Methods**

We conducted a prospective observational multi-center cohort study. The participating centers were 35 primary health care centers (PC) and emergency departments (ED) of 7 hospitals, all clustered into a single area of the Netherlands. Recruitment of consecutive patients who presented with febrile UTI took place from January 2004 to December 2009. The study was approved by the local ethics committees. All participating patients gave written informed consent.

**Inclusion and exclusion criteria**

Inclusion criteria were age of 18 years or above, fever (≥ 38.2^o^C) and/or a history of fever and chills including 24 hours before presentation, at least one symptom of UTI and leukocyturia. Exclusion criteria were present treatment for urolithiasis or hydronephrosis, pregnancy, receipt of hemodialysis or peritoneal dialysis, a history of kidney transplantation or a history of polycystic kidney disease.

**Evaluation**

Baseline patient characteristics were collected by qualified research nurses. Data were collected from the medical record and an interview at the bedside or by telephone using a standardized questionnaire within 24 hours after notification. Collection of data included the predictors that compromise the PRACTICE score. Missing values of categorical variables were considered to indicate the absence of that characteristic. This was applied for diabetes mellitus (n = 2), urinary tract disorder (n = 2) and renal disease (n = 1). In case the medical record reported the respiratory rate to be ‘normal’ or ‘no tachypnea’ (n = 494) this was considered to indicate a respiratory rate < 30/minute. For missing continuous variables the mean of the study population was imputed. This was applied for blood pressure (n = 23), pulse rate (n = 20) and temperature (n = 1).

Blood and urine cultures were taken before commencement of antimicrobial therapy and were performed using standard microbiological methods. All patients were contacted 28-32 days and 84-92 days after enrolment to assess clinical outcome.

**Study outcome**

Our primary outcome was all-cause mortality 30 days after presentation with febrile UTI. Secondary outcomes were need for ICU admission, hospital admission > 10 days, 90-day mortality and a combination of these outcome measures. Survival was assessed using patient or proxy interviews. In case the patient was lost to follow-up, survival was assessed using interview from patient’s primary care physicians and/or hospital chart review and/or local governmental mortality registries. Survival could thus not be assessed with certainty in 12 patients after 30 days and in 17 patients after 90 days. These patients (13 acute uncomplicated pyelonephritis, 4 acute complicated pyelonephritis) were all considered to be alive.

**Statistical analysis**

Descriptive statistics included frequencies, percentages, medians and means. We calculated the area under the receiver operating characteristic curves (AUC) with 95% confidence intervals (CI) to assess a rule’s discriminatory power to predict the outcome. Cut-off values were considered according to sensitivity, specificity, positive and negative predictive values (PPV, NPV) for low- versus high-risk patients. All analyses were performed using SPSS 17.0 (SPPS Inc, Chicago, IL, USA).

**Results**

Of 879 patients screened for eligibility, 787 patients met the inclusion criteria, provided informed consent and were included in the study. 189 (24%) patients were included by PCs and 598 (76%) by EDs. The median age was 67 years and 37% were men. The majority of the patients had comorbidity (Table A).

The results of urine cultures, performed in 742 (94%) patients, were: 421 (54%) *Escherichia coli*, 31 (4%) *Klebsiella* species, 18 (2%) *Proteus* species, 18 (2%) *Pseudomonas aeroginosa*, 16 (2%) *Staphylococcus* species, 13 (2%) *Enterococcus* species, and 26 (4%) other uropathogens; 199 (27%) urine cultures were either sterile or contaminated of which 52% were obtained during UTI treatment. Blood cultures, performed in 743 (94%) patients, revealed bacteremia in 176 (24%) cases; 76% of those grew *E. coli* and 24% other uropathogens.

The median score of the PRACTICE score (range 18-180 points) was 74 (IQR: 48-95). The 30-day mortality rate was 3%. AUC for prediction of 30-day mortality was 0.91 [95% CI: 0.85-0.96], Figure A. Dividing the PRACTICE score into five risk categories, the different clinical outcomes according to risk class are outlined in Table B. The median age across the different PRACTICE score classes were: 32 [IQR 23-40] years for class I, 61 [56-68] for class II, 76 [69-81] for class III, 81 [76-86] for class IV and 86 [80-89] for class V. Across the risk classes the percentages of males were 12, 41, 43, 60 and 53 percent for class I through V respectively. The rates of any co-morbidity were: 29 percent for class I, 51 percent for class II, 81 percent for class III, 91 percent for class IV and 100 percent for V. Mortality, need for ICU admission and duration of hospital stay increased with higher PRACTICE score risk. Though adverse outcomes were exceedingly low for PSI risk class I, II and III, yet a large number of patients within these low risk classes were hospitalized. This suggests that these patients might have been safely treated at home and presumably 40% (class I and II) to 74% (380 of 516 hospitalized patients) (class I, II and III) of the admissions were potentially avoidable. Dichotomizing the PRACTICE score as low risk versus high risk, using a cut-off value of the PRACTICE score ≥ 100 points (class IV and V), resulted in a negative predictive value for predicting 30-day mortality of 100% (95% CI: 99-100%). Because the cut-off point was chosen to identify low-risk patients, the positive predictive value was low: 12% (95% CI: 7-18%). The corresponding sensitivity, specificity and the predictive value for predicting 90-day mortality, need for ICU admission and prolonged hospitalization are outlined in Table C.

**FIGURE AND TABLES**

**Figure A.** The receiver operating characteristics curve of the PRACTICE score for predicting 30 day mortality in adults with febrile UTI.


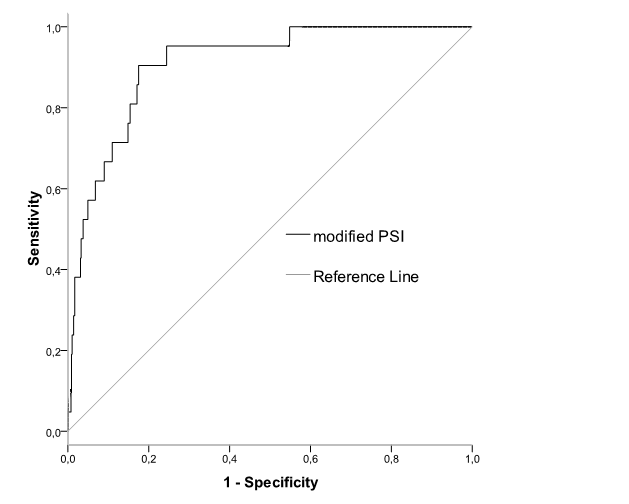


| **Table A.** Patients’ demographics and outcomes | | | |
| --- | --- | --- | --- |
| **Patients (n = 787)** | | | |
| **Site** | | |  |
|  | | Primary health care centers | 189 (24) |
|  | | Emergency departments | 598 (76) |
| **Age** years; median, (IQR) | | | 67 (46-78) |
| **Sex** | | |  |
|  | | Men | 291 (37) |
|  | | Women | 496 (63) |
| **Diagnosis** | | |  |
|  | | Acute uncomplicated UTI/pyelonephritis | 420 (53) |
|  | | Acute complicated UTI/pyelonephritis | 367 (47) |
| **Antimicrobial treatment for UTI** | | | 231 (29) |
| **Urologic history** | | |  |
|  | Present urinary catheter | | 52 (7) |
|  | History of urinary tract disorder | | 215 (27) |
|  | Any history of UTI | | 391 (51) * |
|  | Recurrent UTI | | 189 (25) * |
| **Co-morbidity** | | |  |
| Any | | | 493 (63) |
| Diabetes mellitus | | | 126 (16) |
| Malignancy | | | 84 (11) |
| Heart failure | | | 124 (16) |
| Cerebrovascular disease | | | 105 (13) |
| Renal insufficiency | | | 73 (9) |
| Immunocompromised | | | 107 (14) |
| **Treatment** | | |  |
|  | | Outpatient | 271 (34) |
|  | | Inpatient | 516 (66) |
| **Outcomes** | | |  |
|  | | 30-day mortality | 21 (3) |
|  | | Need for ICU admission | 28 (4) |
|  | | Hospital admission > 10 days | 92 (12) † |
|  | | 90-day mortality | 33 (4) |

Data are presented as n (%) unless otherwise stated. IQR interquartile range, UTI urinary tract infection. Urinary tract disorder: presence of any functional or anatomical abnormality of the urinary tract excluding the presence of a urinary catheter. * UTI history unknown in 21 patients; † 3 missing values.

| **Table B.** Clinical outcome of febrile urinary tract infection according to PRACTICE score risk class. | | | | | | | |
| --- | --- | --- | --- | --- | --- | --- | --- |
|  | |  | | | | |  |
| **PRACTICE score**  **(points)** | | **Class I**  **(<50)** | **Class II**  **(51-75)** | **Class III**  **(76-100)** | **Class IV**  **(101-125)** | **Class V**  **(>125)** | **Total** |
| **No. of patients** | | 211 | 188 | 237 | 105 | 46 | 787 |
|  | |  |  |  |  | |  |
| **Management** | |  |  |  |  |  |  |
|  | Outpatient, No (%) | 104 (49) | 88 (47) | 64 (27) | 11 (11) | 4 (9) | 271 (34) |
|  | Inpatient, No (%) | 107 (51) | 100 (53) | 173 (73) | 94 (89) | 42 (91) | 516 (66) |
|  | |  |  |  |  |  |  |
| **Clinical outcome** | |  |  |  |  |  |  |
| 30-day mortality, % | | 0.0 | 0.5 | 0.8 | 6.7 | 23.9 | 21 (2.7) |
| 90-day mortality, % | | 0.5 | 0.5 | 2.5 | 10.5 | 30.4 | 33 (4.2) |
| ICU admission, % | | 0.9 | 1.1 | 2.5 | 6.7 | 23.9 | 28 (3.6) |
|  | |  |  |  |  | |  |
| **Length of hospital stay** | |  |  |  |  |  |  |
|  | Median no. of days [IQR] | 1 [0-4] | 2 [0-6] | 5 [0-8] | 7 [4-11] | 9 [5-14] | 4 [0-7] |
|  | ≤ 3 days, % | 67.8 | 57.2 | 36.3 | 21.9 | 8.6 | 47.1 |
|  | 4-10 days, % | 30.3 | 34.8 | 46.6 | 54.9 | 45.7 | 39.3 |
|  | > 10 days, % | 1.9 | 8.0 | 17.1 | 30.2 | 45.7 | 13.6 |

ICU intensive care unit

| **Table C.** Predictive value of PRACTICE score ≥ 100 for different clinical outcomes in adults with febrile urinary tract infection | | | | | |
| --- | --- | --- | --- | --- | --- |
| **Clinical outcome**  **(n =787)** | Sensitivity  (95% CI) | Specificity  (95% CI) | NPV  (95% CI) | PPV  (95% CI) | AUC of ROC  (95% CI) |
| 30-day mortality  (n = 21) | 0.86  (0.63-0.96) | 0.83  (0.79-0.85) | 1.00  (0.99-1.00) | 0.12 (0.07-0.18) | 0.84  (0.75-0.93) |
| 90-day mortality  (n = 33) | 0.76  (0.57-0.88) | 0.83  (0.80-0.86) | 0.99  (0.97-0.99 | 0.17 (0.11-0.24) | 0.80  (0.71-0.88) |
| 30-day mortality and/or ICU admission (n = 41) | 0.71  (0.54-0.83) | 0.84  (0.81-0.86) | 0.98  (0.97-0.99) | 0.19 (0.13-0.27) | 0.77  (0.69-0.86) |
| 30-day mortality and/or ICU admission and/or > 10 days hospitalization  (n = 122) | 0.48  (0.39-0.57) | 0.86  (0.83-0.89) | 0.90  (0.87-0.92) | 0.39 (0.31-0.47) | 0.67  (0.62-0.73) |
| CI: confidence interval; NPV: negative predictive value; PPV: positive predictive value; AUC of ROC; area under the curve of receiver operating characteristic; ICU: intensive care unit. | | | | | |
